# The X chromosome and male infertility

**DOI:** 10.1007/s00439-019-02101-w

**Published:** 2019-12-24

**Authors:** Matthias Vockel, Antoni Riera-Escamilla, Frank Tüttelmann, Csilla Krausz

**Affiliations:** 1 Institute of Human Genetics, University of Münster, Vesaliusweg 12-14, 48149 Münster, Germany; 2 Andrology Department, Fundació Puigvert, Universitat Autònoma de Barcelona, Instituto de Investigaciones Biomédicas Sant Pau (IIB- Sant Pau), Barcelona, Catalonia Spain; 3 Department of Experimental and Clinical Biomedical Sciences “Mario Serio”, Centre of Excellence DeNothe, University of Florence, Viale PIeraccini, 6, 50139 Florence, Italy

## Abstract

The X chromosome is a key player in germ cell development, as has been highlighted for males in previous studies revealing that the mammalian X chromosome is enriched in genes expressed in early spermatogenesis. In this review, we focus on the X chromosome’s unique biology as associated with human male infertility. Male infertility is most commonly caused by spermatogenic defects to which X chromosome dosage is closely linked; for example, any supernumerary X chromosome as in Klinefelter syndrome will lead to male infertility. Furthermore, because males normally only have a single X chromosome and because X-linked genetic anomalies are generally only present in a single copy in males, any loss-of-function mutations in single-copy X-chromosomal genes cannot be compensated by a normal allele. These features make X-linked genes particularly attractive for studying male spermatogenic failure. However, to date, only very few genetic causes have been identified as being definitively responsible for male infertility in humans. Although genetic studies of germ cell-enriched X-chromosomal genes in mice suggest a role of certain human orthologs in infertile men, these genes in mice and humans have striking evolutionary differences. Furthermore, the complexity and highly repetitive structure of the X chromosome hinder the mutational analysis of X-linked genes in humans. Therefore, we conclude that additional methodological approaches are urgently warranted to advance our understanding of the genetics of X-linked male infertility.

## Introduction

Infertility is a frequent condition affecting one out of six couples in Western countries (Krausz and Riera-Escamilla 2018). Male factors contribute to infertility in about 50% of cases and can be classified into four major etiological categories: (i) hypothalamic–pituitary axis dysfunction; (ii) quantitative alterations of spermatogenesis; (iii) qualitative alterations of spermatogenesis; and (iv) ductal obstruction/dysfunction (Tournaye et al. 2016). Known genetic factors are present in each of these etiological categories, and, depending on the severity of the spermatogenic impairment, they account for about 10–20% of cases. With the advent of large-scale sequencing strategies (especially exome analysis by next-generation sequencing, or NGS), more and more novel genes have been reported to be linked with male infertility. However, the majority of these genes still lack a definitive gene–disease relationship (Oud et al. 2019). Further, about 40% of quantitative spermatogenic impairment cases cannot be causally diagnosed and are instead referred to as unexplained or idiopathic infertility; a large proportion of these cases are likely caused by yet unknown genetic factors.

Both sex chromosomes in males (X and Y) are enriched in genes specifically or overexpressed in the testis (Skaletsky et al. 2003; Mueller et al. 2008). Moreover, in males, de novo or rare mutations in these chromosomes may have a direct phenotypic effect because there is no second compensatory X or Y allele. The role of Y chromosome-linked deletions in male infertility has been well understood since 1996 (Vogt et al. 1996). Here, the removal of genes belonging to the so-called azoospermia factor (AZF) region causes azoospermia or oligozoospermia (Krausz et al. 2014; Krausz and Casamonti 2017). While screening for Y chromosome deletions is a fundamental part of the diagnostic workup of severe oligo/azoospermic men, only two X chromosome-linked genes are routinely tested in selected pathological conditions (*ANOS1* and *AR*). Thanks to high-throughput approaches such as whole exome sequencing (WES) and comparative genomic hybridization (CGH) arrays, novel X-linked gene mutations (e.g., *TEX11* and *ADGRG2*) have been discovered as causative factors of different infertile phenotypes.

In light of these findings, in this review we will discuss (i) X-linked genes and male fitness from an evolutionary point of view; (ii) the clinical consequences of X-linked aneuploidies and deletions; (iii) the role of X-linked gene mutations in male infertility.

## Structure and function of the X chromosome

While both sex chromosomes are derived from a pair of autosomes around 300 million years ago, most ancestral functional elements have been only conserved on the X chromosome (Ross et al. 2005). The sex chromosome’s size also differs dramatically, and while the X chromosome has remained relatively stable in magnitude with around 155 Mb, the Y chromosome decreased to around 55 Mb. The X chromosome contains about 800 (human) to 850 (murine) protein-coding genes (Mueller et al. 2013), for which a disproportionately high number of inherited diseases were documented. The unique gene composition of the X chromosome also allows balanced gene dosage compensation through a well-regulated spatial and temporal expression of X-linked genes (Deng et al. 2014).

The X chromosome was previously considered the most stable chromosome of the genome (Ohno 1967) and was largely perceived as a “female” counterpart to the “male” Y chromosome. But, to the contrary, it turns out that the X chromosome is in a current state of rapid evolution toward a specialized role for sperm production.

The hemizygosity in males allows for unique evolutionary patterns of X-linked genes for male sexual fitness and can favor positive selection of advantageous variants (Vicoso and Charlesworth 2006). Accordingly, the X chromosome is enriched for sex-biased (Khil et al. 2004) and spermatogonia-expressed (Wang et al. 2001) genes. However, X-linked genes are thought to be silenced during male meiosis by a mechanism known as meiotic sex chromosome inactivation (Turner 2007). To compensate for this inactivation, large gene families with multiple copies have accumulated on the X chromosome (Warburton et al. 2004), and these homologous palindromic repeats are highly expressed in spermatids (Mueller et al. 2008). An example of an X-chromosomal multigene family is the large superfamily called cancer testis antigens (CTA), in which members are arranged into complex direct and palindromic repeats. As has been estimated, CTA families account for around 10% of X-linked genes (Simpson et al. 2005; Almeida et al. 2009), and they can be divided into the *MAGE* multigene family and the *NY*-*ESO*-*1* family in region Xq24-q28 and the *SSX* family and *GAGE/PAGE/XAGE* families in region Xp11 (Zendman et al. 2003). Most of the CTA genes are expressed during spermatogenesis, but their function in both germline tissues and tumors remains poorly understood (Fratta et al. 2011).

It has been speculated that multicopy regions on the X chromosome may have an important function for germ cell survival, e.g., through dosage compensation. In this regard, homologous repeats could self-pair during male meiosis such that they would potentially be protected from unpaired chromosome inactivation, as has been suggested for the Y chromosome (Skaletsky et al. 2003). However, a recent study implies that copy number expansions of palindromic genes might not be essential for spermatogenesis, as reduced ampliconic gene expression following deletion of large X-linked palindromes did not reduce fertility in male mice (Kruger et al. 2018).

## X chromosome-linked genes and male fitness: an evolutionary perspective

**Fig. 1:**
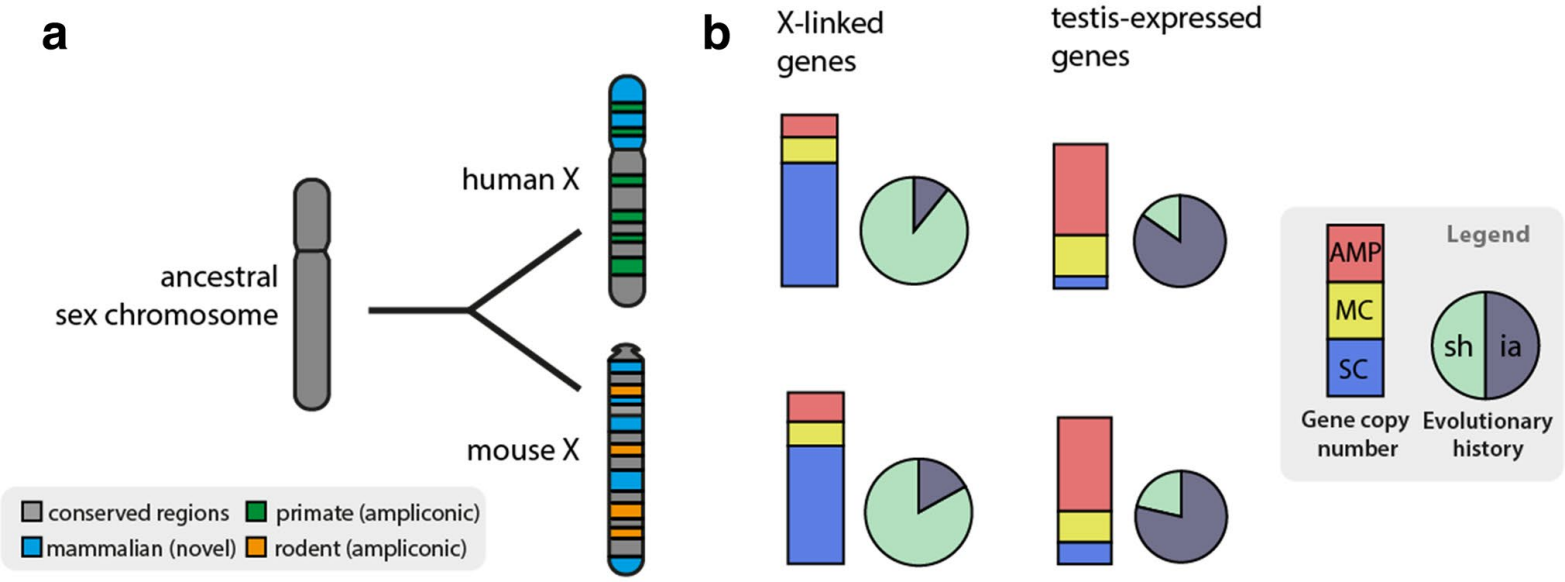
Evolution of mammalian X chromosomes. Contrary to Ohno’s law, the gene content of the X chromosomes differs significantly within the mammalian lineage. **a** Schematics of the variability of X-linked genes between rodents and primates. Colors denote the divergent evolutionary origins of X‑linked genes. Gray: conserved ancestral regions (corresponds to the long arm of the human X, and this region is rearranged in mice); Blue: acquired gene content in mammals (short arm of human X, region rearranged in mice); green and orange: independently acquired ampliconic genes in humans and mice, respectively (positions correspond to arbitrary genomic locations). **b** Depicted diagrams show a comparison of all X-linked (*n* = 800 human, 853 mouse) and predominantly testis-expressed genes (expression patterns of eight human tissues and three mouse tissues) in humans and mice. Bar graphs: percentages of protein-coding X-linked genes categorized as single copy (SC, blue), multicopy (MC, yellow) or ampliconic (AMP, red). Pie charts: percentages of X-linked genes that are shared (sh, light green) in both species or are independently acquired (ia, dark blue) after the divergence of humans and mice. Most ampliconic X-linked genes have been independently acquired in primates and rodents and are predominantly expressed in testes (data adopted from Bellott et al. 2010; Mueller et al. 2013; Deng et al. 2014)

More than 50 years ago, it was postulated that the X chromosome is in an evolutionary stable state, and X-linked genes should differ little between placental mammals (Ohno 1967). This hypothesis, called ‘Ohno’s law’, is supported by several comparative mammalian studies (e.g., Murphy et al. 1999; Kuroiwa et al. 2001; Raudsepp et al. 2004; Ross et al. 2005). Later though, it was shown that X chromosomes have undergone rapid evolutionary changes, and accumulation of ampliconic genes has occurred individually during mammalian evolution (Bellott et al. 2010; Mueller et al. 2013). This comparison of multicopy genes in different lineages has offered important insights into the mammalian evolution of X-linked genes. While most of the single-copy genes on the mouse and human X chromosomes are conserved between these species, approximately 340 genes are not shared between the two species, and most of these unshared genes are found in ampliconic regions (Fig. 1). About 10% of the human and 16% of mouse X-linked genes, respectively, have been independently acquired since their divergence from a common ancestor. In contrast to the shared, single-copy genes, these ampliconic genes are notable exceptions to the postulated Ohno’s law. Expression analyses further revealed that these ampliconic genes are active almost exclusively in testicular germ cells, but not in other male tissues or female tissues. As such, they are likely to contribute to sperm production (Mueller et al. 2013). Although the physiological functions of most independently acquired, ampliconic genes are still unclear, their rapid evolution suggests a strong positive selection that may have contributed to speciation.

It is possible that these genes have significant roles in diseases related to infertility, but the use of model systems for these genes is complicated because they are not conserved in mice. This caveat seems to apply to all sex-linked genes, as a recent study has suggested that sex bias in overall gene expression also arose recently in mammalian evolution and, hence, could be species specific, which might prevent results on molecular variation from being translated from animal models to human reproduction research (Naqvi et al. 2019). Furthermore, sex chromosomes evolve functional specialization by accumulation of sexually antagonistic as well as sex-biased genes (Vicoso and Charlesworth 2006; Parsch and Ellegren 2013). The significant enrichment for testis-expressed genes on the X chromosome and the differential expression of sex-biased genes imply that sexual conflict has profound consequences on gene content evolution of X chromosomes in mammals (Gurbich and Bachtrog 2008; Larson et al. 2018).

## The consequences of too much and too little X chromosome

### Too much X: Klinefelter syndrome

Klinefelter syndrome (KS) is a chromosomal condition that was first described in 1942 (Klinefelter et al. 1942). It was not until 1959 when Jacobs and Strong (1959) determined that it was caused by the presence of an extra X chromosome. Various genotypes are associated with this condition, the most common being 47,XXY in up to 80–90% of cases, while in approximately 10% of cases the genotype is a mosaic form of 47,XXY/46,XY. Other rare variants are 48,XXXY; 48,XXYY; 49,XXXXY; 49,XXXYY; and 49,XXYYY and are associated with more severe phenotypes (Visootsak et al. 2001; Tartaglia et al. 2008; Ottesen et al. 2010). KS shows an estimated frequency of 1:600 in the general population, whereas in patients with non-obstructive azoospermia it is as high as 15%. In adults, the diagnosis is mainly made because of infertility and/or sexual dysfunction. The reproductive phenotype of these individuals includes small, firm testes with hyalinization of seminiferous tubules with consequent spermatogenic failure. In fact, over 90% of KS patients are azoospermic, and the remaining show crypto/severe oligozoospermia. Despite that spontaneous puberty is observed in these patients; the majority of them present signs of androgen deficiency, which range from hypogonadism with gynecomastia and eunuchoid proportions to variable levels of undervirilization.

Natural pregnancies have been reported mostly in mosaic KS cases. For the majority of non-mosaic 47,XXY KS men, conception of a biological child is only possible through testicular sperm extraction (TESE), either conventional TESE or microTESE combined with intracytoplasmic sperm injection (ICSI) (Corona et al. 2017). The success rate of sperm retrieval varies in different laboratories, but according to a recent meta-analysis the mean sperm retrieval rate (SRR) is about 44% (Corona et al. 2017). Although an accurate prediction of SRR is not possible, age seems to be a relevant prognostic parameter, and KS patients under 30 years old show a higher success rate (Rohayem et al. 2015). Hence, an early diagnosis of this syndrome is clearly relevant, since it would allow both the preservation of fertility and the introduction of preventive therapies to avoid or to better control associated co-morbidities. In fact, besides reproductive/sexual problems, KS patients may present a series of co-morbidities such as metabolic syndrome, autoimmune diseases, osteoporosis, breast and germ cell tumors, venous thromboembolisms and cognitive/psychiatric disturbances (Gravholt et al. 2018 and references therein).

What underlies the heterogeneous phenotypic presentation is still not clear, but is probably related to various factors (Tüttelmann and Gromoll 2010; Zitzmann et al. 2015). For instance, the proposed phenotypic modulators include parental origin of the extra X chromosome, the androgen receptor CAG repeat length, gene dosage dysregulation, and/or epigenetic events. First, the parental origin of the supernumerary X chromosome has only been addressed by few studies reporting a potential effect on impaired speech/motor development (Stemkens et al. 2006), later onset of puberty (Wikstrom et al. 2006) and schizotypal traits (Bruining et al. 2010). While impaired speech/motor developmental problems and later onset of puberty were significantly associated with paternal origin of the X chromosome, significantly higher scores on schizotypal traits were associated with maternal origin.

Next, regarding the CAG repeats in the androgen receptor (*AR*) gene, the number modulates its transcriptional activity in vitro (Gao et al. 1996), and an increased number of repeats was positively correlated with height, arm span and both arm and leg length in KS patients (Zitzmann et al. 2004; Bojesen et al. 2011; Chang et al. 2015). On the other hand, data on the (CAG)n polymorphism in relation to testicular volume, gynecomastia, lipid metabolism and bone-related parameters are still controversial (Gravholt et al. 2018 and references therein).

Regarding dosage dysregulation, women have two X chromosomes, while men only have one. Thus, to compensate for the difference in gene dosage, in females one of the two X chromosomes is inactivated. However, a small portion of X-chromosomal genes (~ 10–15%) escape inactivation, among it the PAR genes. In this regard, the overdosage of *SHOX* has been demonstrated to be associated with the typical excess height of KS patients. Apart from *SHOX*, other PAR genes such as *CD99* and *CSF2RA* were found differentially expressed in the KS group vs. male controls (Zitzmann et al. 2015). The overexpression of these two genes could contribute to the development of some of the typical co-morbidities of KS [overexpression of *CD99* was associated with inflammatory cytokines, whereas *CSF2RA* with insulin resistance, waist circumference, and concentrations of the procoagulatory substance PAI-1 and cytokines (Zitzmann et al. 2015)].

Finally, regarding epigenetic events, the supernumerary X chromosome in KS has an interesting effect on DNA methylation. Methylome studies in blood and brain samples have shown differences between KS patients and 46,XY males (Viana et al. 2014; Sharma et al. 2015; Wan et al. 2015; Skakkebaek et al. 2018). Unfortunately, only few overlapping differentially methylated regions (DMRs) have been observed between studies, which could be in part explained by differences in the type of sample analyzed (blood vs brain) and the resolution of the arrays (Illumina 27 K vs Illumina450K) (Skakkebaek et al. 2018 and references therein). Gene set enrichment analysis of the observed DMRs showed an overrepresentation of terms relevant to the KS phenotype such as diabetes, obesity, height, coronary and arterial disease, hypercholesterolemia, gingivitis and periodontitis, bone mineral density, cancer (breast, prostate, colon, pancreas, leukemia, lymphoma), connective tissue diseases, infection and inflammation and others (Skakkebaek et al. 2018). However, it is still unknown whether these epigenetic alterations are responsible for the KS co-morbidities through altering gene expression regulation. In fact, only a moderate correlation between epigenetic alterations and some of the phenotype-associated gene expression levels (*BOLA1*, *DDX43*, *ZBTB44* and *KDM5C*) has been observed (Skakkebaek et al. 2018). Further studies on different tissue samples are needed to elucidate the biological basis for the heterogeneous phenotypic presentation of KS.

### Too little X: X-linked deletions

Copy number variants (CNVs) are a type of structural variant involving alterations in the number of copies of specific regions of DNA (often defined as above 1 kb in length) in comparison with a reference genome (Feuk et al. 2006). The functional consequence of a CNV can be difficult to predict and depends on the exact position of the CNV breakpoint, i.e., the region where a fragment was inserted (gain/duplication) or lost (loss/deletion). CNVs may affect gene function directly or indirectly. A direct effect through the involvement of coding sequences may be the consequence of the CNV (i) altering the copy number of dosage-sensitive genes; (ii) disrupting the gene-coding sequence, as partial gain or deletion of coding sequences can produce different alleles, including both loss and gain of function; or (iii) producing chimeric proteins when CNV breakpoints lie within two different genes, leading to the fusion of two partial coding regions. An indirect effect may occur when CNVs lie in non-coding regions and disrupt the function of genes through the deletion or transposition of critical regulatory elements, such as promoters, enhancers and silencers (Hurles et al. 2008).

A number of CNVs generate alleles with a pathogenic effect in andrology; the best examples of such a clear-cut effect are the Y-chromosomal AZF deletions (Krausz et al. 2014; Krausz and Casamonti 2017). Comparative genomic hybridization (CGH) arrays (whole genome or X chromosome specific) have been employed to analyze patients with quantitative spermatogenic disturbances in five laboratories (Tüttelmann et al. 2011; Krausz et al. 2012; Stouffs et al. 2012; Lopes et al. 2013; Yatsenko et al. 2015), and four of them provide information about X-linked CNVs (Tüttelmann et al. 2011; Krausz et al. 2012; Lopes et al. 2013; Yatsenko et al. 2015). Comparing the raw data of these studies, only few partially overlapping CNVs have been observed, which could be in part explained by differences in the resolution of the arrays (Krausz et al. 2015). The only consistent finding of the available studies is that there is a significantly higher ‘burden’ of deletions, particularly on the X chromosome, in infertile men versus normozoospermic controls. The observed higher burden of X-linked deletions has been hypothesized to be an expression of a generalized genomic instability, which may also lead to general health problems. This hypothesis would be in line with the epidemiological observations showing a link between altered spermatogenic function and a higher incidence of morbidity (including cancer) and lower life expectancy (Jensen et al. 2009; Salonia et al. 2009; Eisenberg et al. 2014).

Besides the CNV burden, CGH arrays have allowed researchers to identify a few X-linked deletions with a possible role in male infertility (Krausz et al. 2015 and references therein). Only one potential clinically relevant X chromosome-linked deletion has been analyzed in several studies. CNV67 was first described as patient specific in 1.1% (7/627) of infertile men from Spain and Italy (Lo Giacco et al. 2014). A subsequent study in a Mediterranean population confirmed the role of the CNV67 in male infertility, since it was found in 1.2% of infertile men but not in normozoospermic controls (Pinho et al. 2019). The CNV67 has also been analyzed in the Chinese population with controversial results. One study reported that 6/884 infertile patients carried the deletion, whereas it was not found in any of the 838 controls from Sichuan Province in southwestern China (Shen et al. 2017). On the contrary, Ma et al. (2017) reported the prevalence of the CNV67 to be about 3% in fertile controls, but the controls had unknown sperm counts and belonged to a specific geographic area (the Guandong population). The exact mechanism by which CNV67 may alter spermatogenesis is still unknown. Studies have suggested that the CNV67 might cause a partial deletion of the proximal copy of the *MAGEA9B* gene, which is a CTA specifically expressed in the testis and in some tumors (Shen et al. 2017). In summary, further studies in multiple populations including fertile controls with known sperm counts are needed to elucidate the clinical relevance of this CNV.

## X chromosome-linked gene mutations in male infertility

Many X-linked genes are promising candidates as causes for infertility owing to their testicular expression and expected involvement in spermatogenesis. The advances in high-throughput sequencing approaches have allowed for the identification of additional genetic factors in azoospermic patients either by targeted sequencing or exome sequencing. However, most studies have not been replicated so far, and the functional roles of most genes are still ill-defined. Furthermore, the repetitive nature of the X chromosome can complicate the detection of deleterious variants. Although several candidate genes have been postulated, the number of promising or definitively causative genes for male infertility remains low when assessed in a standardized way (Smith et al. 2017), as has recently been done by Oud and collogues (Oud et al. 2019); see Table 1.

**Table 1:** Overview of disease-associated X-linked genes with testis-specific expression in mice and humans

Gene	Semen/testicular phenotype	Proposed functions	Mouse phenotype	Publications	Level of evidence
Phenotype: isolated spermatogenic failure					
*AR*	Oligozoospermia, azoospermia	Hormone activated transcription factor	Infertile; partial androgen insensitivity syndrome	Hiort et al. (2000), Ferlin et al. (2006)	Definitive
*TEX11*	Non-obstructive azoospermia, meiotic arrest	Meiotic DNA recombination	Infertile; meiotic arrest	Yang et al. (2015a), Yatsenko et al. (2015)	Strong
*MAGEB4*	Non-obstructive azoospermia	Germ cell-specific mitosis	No mouse model	Okutman et al. (2017)	Limited
*HAUS7*	Severe oligozoospermia	Meiotic chromosome alignment	No mouse model	Li et al. (2018)	Limited
*USP26*	Oligozoospermia, azoospermia	Deubiquitinating peptidase	Fertile	Paduch et al. (2005)	Limited
*RHOXF1/2*	Oligozoospermia, severe	Homeodomain transcription factor	No mouse model	Borgmann et al. (2016)	No evidence
*TAF7L*	Non-obstructive azoospermia	TFIID transcription factor	Complete spermatogenesis; abnormal sperm number/morphology	Akinloye et al. (2007)	No evidence
Phenotype: obstructive azoospermia/CBAVD					
*ADGRG2*	Congenital bilateral absence of the vas deferens (CBAVD)	G-protein-dependent fluid homeostasis	Infertile; obstructive	Patat et al. (2016)	Strong
*CLDN2*	Obstructive azoospermia	BTB tight junction formation	Fertile	Askari et al. (2019)	Limited^a^
Phenotype: astheno-/teratozoospermia					
*PIH1D3*	Asthenozoospermia	Flagella dynein assembly	Infertile; mild PCD	Paff et al. (2017), Olcese et al. (2017)	Strong
*AKAP4*	Asthenozoospermia, teratozoospermia	PKA binding in sperm flagellum	Infertile; multiple morphological abnormalities of the sperm flagella	Baccetti et al. (2005), Visser et al. (2011)	Limited
*FATE1*	Oligoasthenozoospermia	SF1-dependent mitochondrial regulation	No mouse model	Olesen et al. (2003)	No evidence
Phenotype: Kallmann syndrome					
*ANOS1*	Oligozoospermia, azoospermia	GnRH neuronal migration	Infertile; hypogonadotropic hypogonadism	Gonçalves et al. (2017)	Definitive

^a^Not in Oud et al. (2019) because published subsequently. Assessment according to same criteria by MV and FT independently

## Non-obstructive azoospermia (NOA)

### AR

The androgen receptor (*AR*) remains the best studied X-linked gene so far in the etiology of male infertility. The AR is critical for diverse developmental processes and generally functions as a DNA-binding transcription factor. Upon binding of steroid hormone ligands in the cytoplasm, the receptor translocates into the nucleus and then stimulates the expression of androgen-responsive genes. The phenotype of patients with mutations in AR is highly variable and varies from complete androgen insensitivity syndrome (CAIS) with sex reversal to mild symptoms (Brinkmann 2001). Patients with mild androgen insensitivity syndrome (MAIS) in particular can have normal genitalia and isolated male infertility. Variants detected in infertile patients are mainly missense mutations affecting the transactivation potential of the AR (Goglia et al. 2011; O’Hara and Smith 2015). However, of the great number of reported AR mutations (Gottlieb et al. 2012), only a small number have been clearly associated with azoospermia or oligozoospermia. Thus, *AR* mutations can be considered a rare cause of male infertility (Hiort et al. 2000; Ferlin et al. 2006).

Furthermore, the *AR* gene contains a polymorphic CAG repeat in exon 1. Repeats longer than 35 are associated with spinal and bulbar muscular atrophy (SBMA) and prostate cancer (McCrea et al. 2016; Cortes and La Spada 2018). Besides, the length of the polymorphic CAG repeat encoding a polyglutamine stretch (8–35 in length) in the AR N-terminal transactivation domain has been linked to male infertility. The number of CAG repeats is inversely correlated with transcriptional activity in vitro (Tut et al. 1997; Ferlin et al. 2004). The association between the AR-CAG repeat and male infertility has been intensively studied, but effects seem to be small and are still debated. Longer CAG repeat lengths may be a risk factor for male infertility, although population studies with variation detection in healthy controls argue against a strong association with unexplained male infertility, as concluded from large meta-analyses (Davis-Dao et al. 2007; Xiao et al. 2016). The transactivation domain of the AR comprises a further polyglycine tract (10–30 in length) encoded by a repeat consisting of GGN trinucleotides. However, the functional effect of the polyglycine tract remains controversial (Jääskeläinen 2012; Grigorova et al. 2017).

Apart from *AR*, additional X-chromosomal genes have been postulated to be causative for male infertility, including *TEX11*, *MAGEB4*, *RHOX* and *TAF7L*.

### TEX11

*TEX11* encodes a protein that regulates DNA recombination and formation of chromosome synapses and crossovers (Adelman and Petrini 2008). The gene is highly expressed in late spermatocytes and in spermatids in mice (Wang et al. 2001), and disruption of *Tex11* in knock-out mice causes meiotic arrest and results in azoospermia (Yang et al. 2008). Recently, an intragenic *TEX11* deletion removing exons 10–12 was identified by high-resolution X-specific array-CGH in two infertile patients with NOA due to meiotic arrest and mixed testicular atrophy, respectively (Yatsenko et al. 2015). Further analysis by targeted sequencing identified further *TEX11* variants of which loss-of-function (LoF) mutations were associated with meiotic arrest, while the role of missense variants remains less clear (Yatsenko et al. 2015; Yang et al. 2015a). Together, these data provide strong evidence that LoF variants in *TEX11* cause non-obstructive azoospermia due to meiotic arrest also in human males.

### MAGEB4

*MAGEB4* is a type I melanoma antigen gene from the CTA family specifically expressed during germ cell differentiation with a yet-unclear function, although a role in murine germ cell-specific mitosis has been postulated (Osterlund et al. 2000). This gene is part of a multicopy gene cluster of type I melanoma antigens (Lee and Potts 2017). In a single consanguineous family, a stop-loss variant was segregated with azoospermia and oligozoospermia (Okutman et al. 2017). The evidence that this gene plays a causative role in NOA is currently very limited, as (i) the functional relevance of the described stop-loss variant is still unclear; (ii) no other variants in *MAGEB4* have been identified so far; and iii) the role of other homologous MAGE family members remains elusive.

### HAUS7

*HAUS7* encodes a subunit of the human Augmin protein complex (Goshima et al. 2008) with a proposed function in chromosome alignment during meiosis. While a single *HAUS7* variant has been described in a familial case of severe oligozoospermia (Li et al. 2018), its role in NOA pathology remains to be determined.

### USP26

USP26 is a deubiquitylating enzyme with a transcription pattern restricted to the male germline (Wang et al. 2001). Several variants of unknown clinical significance were reported in azoospermic patients (Paduch et al. 2005; Ma et al. 2016; Luddi et al. 2016). However, reported missense and nonsense *USP26* variants were also detected in fertile controls (Ravel et al. 2006; Christensen et al. 2008; Shi et al. 2011), and the functional impact of missense variants on enzyme activity appears to be low (Zhang et al. 2015; Liu et al. 2018). In vivo analysis of knock-out mice suggests that *Usp26* is dispensable for mouse gametogenesis (Felipe-Medina et al. 2019). Hence, convincing evidence for an association of *USP26* variants with male infertility is currently lacking.

### RHOX

*RHOX* genes belong to a large multicopy homeobox gene cluster (Maclean et al. 2005) and are expressed in male germ cells (Song et al. 2013). In severe oligozoospermic patients, variants were described in the genes *RHOXF1* and *RHOXF2* (Borgmann et al. 2016). However, it remains unclear whether variants in a single *RHOX* gene could be causative for NOA, as other members of this large gene cluster might have redundant roles (MacLean and Wilkinson 2010). Further exploration of these ampliconic regions is necessary for a conclusive evaluation of pathogenicity.

### TAF7L

The transcription factor *TAF7L* is expressed during meiosis with a dynamically regulated intracellular localization (Pointud et al. 2003). Although *Taf7l* knock-out mice normally complete meiosis, they show reduced sperm production and abnormal sperm morphology (Cheng et al. 2007). Variants in *TAF7L* were described as risk factors for spermatogenic failure (Akinloye et al. 2007), although most variants were also found in fertile controls (Stouffs et al. 2006). Taken together, the disease association of *TAF7L* variants with azoospermia remains with no evidence.

## Obstructive azoospermia

### ADGRG2

Obstructive azoospermia can be caused by congenital bilateral absence of the vas deferens (CBAVD). In most CBAVD cases, recessive mutations are identified in the cystic fibrosis transmembrane conductance regulator gene (*CFTR*), and *CFTR* analysis by sequencing and screening for large rearrangements allows for a mutation detection rate of about 87% in CBAVD patients (Ratbi et al. 2007). By exome sequencing of *CFTR*-negative CBAVD patients, LoF mutations were recently identified in the X-linked *ADGRG2* gene (Patat et al. 2016), which encodes an adhesion G protein-coupled receptor that is specifically expressed in the epididymis and efferent ducts (Obermann et al. 2003). Subsequently, further truncating and missense variants in *ADGRG2* have been described (Yang et al. 2017; Yuan et al. 2019; Pagin et al. 2019). In accordance with human data, previous studies have already demonstrated that *Adgrg2*-knock-out male mice develop obstructive infertility (Davies et al. 2004). These studies established *ADGRG2* as a novel gene responsible for a specific subtype of obstructive azoospermia. The molecular mechanism of X-linked CBAVD remains uncertain, although a regulation of fluid reabsorption by G-protein-dependent CFTR coupling and β-arrestin-dependent receptor internalization has been postulated (Zhang et al. 2018; Azimzadeh et al. 2019).

### CLDN2

Recently, a *CLDN2* missense variant was described in a family with several male relatives affected by obstructive azoospermia (Askari et al. 2019). The encoded protein Claudin-2 is a paracellular transmembrane protein, and the authors proposed a gain-of-function effect on the blood–epididymis barrier by the missense variant. However, *Cldn2*-knock-out mice are fertile, and CLDN2 was shown to be expressed in leaky epithelia, in contrast to barrier-forming claudins like CLDN1/4 (Muto et al. 2010). It remains to be shown whether *CLDN2* variants can be responsible for obstructive azoospermia, e.g., by influencing the paracellular water transport in the epididymis.

## Teratozoospermia and asthenozoospermia

### PIH1D3

*PIH1D3* (also known as *CXorf41*) encodes a protein which plays a role in preassembly of both outer (ODA) and inner (IDA) dynein arms of cilia and sperm flagella (Dong et al. 2014; Paff et al. 2017; Olcese et al. 2017). Point mutations and complete *PIH1D3* gene deletions have been reported in patients affected by primary ciliary dyskinesia (PCD) (Paff et al. 2017; Olcese et al. 2017). PCD is a multisystemic disorder characterized by chronic respiratory tract infections, abnormally positioned internal organs and asthenozoospermia due to motility defects of cilia and flagella (Knowles et al. 2016). PCD can be caused by a number of genes, and transmission can be autosomal dominant, recessive or X-linked, with *PIH1D3* as the only validated X-linked PCD gene. For *PIH1D3* carriers, ICSI is an option. However, it should be noted that they are at risk for transmission of the entire spectrum of symptoms to the female offspring in case their partner is a heterozygous carrier.

### AKAP4

*AKAP4* encodes a major structural component of sperm fibrous sheath (Turner et al. 1998). Intragenic deletions of the *AKAP3* and *AKAP4* genes were first described in a patient with total sperm immotility due to dysplasia of the fibrous sheath (DFS) (Baccetti et al. 2005). In 2011, an additional patient with DFS was reported carrying a hemizygous missense variant in *AKAP4* (Visser et al. 2011). The Akap4-defficient mice resemble the asthenozoospermic phenotype (Fang et al. 2019) observed in men. However, apart from these two case reports, *AKAP4* mutations were not found in other cohorts of DFS patients, indicating currently limited evidence for this gene defect (Turner et al. 2001a, b; Yang et al. 2015b; Pereira et al. 2015).

### FATE1

*FATE1* belongs to the CTA family, and in 2003 it was sequenced in a cohort of 144 randomly selected infertile patients (Olesen et al. 2003). The authors reported two missense variants in two unrelated asthenozoospermic men. However, for one of the carriers a family study revealed that the variant was compatible with fertility; for the other one, the current bioinformatic tools predict the variant as benign. Therefore, given that no more cases have been reported since 2003, it is highly unlikely that this gene plays an important role in male infertility.

## Hypogonadotropic hypogonadism

### ANOS1

*ANOS1* (Xp22.31), previously called *KAL1*, encodes the anosmin-1 glycoprotein which is involved in the migration of GnRH neurons. *ANOS1* point mutations and gene deletions are the most common cause of Kallmann syndrome, a rare endocrine disease characterized by both congenital hypogonadotropic hypogonadism (cHH) and hyposmia/anosmia. *ANOS1* mutations and deletions may explain the phenotype in about 5–10% of cases (Balasubramanian and Crowley 1993); in addition to *ANOS1*, mutations in more than 20 autosomal genes can also lead to Kallmann syndrome.

The reproductive phenotype of patients affected by cHH may range from the complete form with absent puberty (usually associated with cryptorchidism, micropenis and gynecomastia) to late-onset HH (Krausz et al. 2018 and references therein). Other non-reproductive and non-olfactory alterations described in *KAL1* mutation carriers are bimanual synkinesia (in about 80% of cases), unilateral renal dysgenesis (30%), arched palate, tooth agenesis, hearing impairment, pes cavus and ptosis, among others (Balasubramanian and Crowley 1993; Dode and Hardelin 2009). Spermatogenesis is inducible with the administration of gonadotrophins, hence cHH patients may generate their own biological children either through natural conception or assisted reproductive techniques. Preimplantation genetic testing in syndromic forms is advisable to prevent the transmission of the mutation.

## Conclusions

Currently, validated X chromosome-linked monogenic causes of male infertility are surprisingly rare. From a total of eight genes studied in men with idiopathic oligo/azoospermia, only *AR* and *TEX11* reach sufficiently high clinical evidence for inclusion in diagnostic testing. On the other hand, targeted studies on highly specific infertile phenotypes have successfully identified a novel gene for congenital absence of vas deferens (*ADGRG2)* and for complete asthenozoospermia due to primary ciliary dyskinesia (*PIH1D3*).

Although the X chromosome contains several structural elements which may predispose it to AZF-like deletions, only one deletion, CNV67, has been the object of multiple validation studies. This deletion affects *MAGEA9*, an ampliconic gene belonging to one of the CTA gene families. Similar to CNV67, the majority of X-linked deletions and duplications affect or remove CTA genes. These genes were independently acquired on the human X chromosome and are exclusively expressed in the testis in physiological conditions. Based on their expression profile and their predicted involvement in cell proliferation (expression in various cancers), these genes are predicted to play a role in spermatogenesis. However, the interpretation of mutations in CTA genes is largely limited by their multicopy nature (implying possible compensation by the retained copies) and the lack of model animals (due to their exclusive presence in the human genome). Future, targeted studies are warranted on this specific group of peculiar genes.

The paucity of clinically relevant X-linked anomalies in oligo/azoospermia is in sharp contrast with the predicted specialization of this chromosome in male reproductive fitness. Reasons for these disappointing results can be related to the fact that the majority of studies focusing on quantitative impairment of spermatogenesis were based on Sanger re-sequencing of a few candidate genes. Ongoing exome analyses or whole X chromosome gene panel studies in large patient cohorts are likely to reveal novel X-linked genetic factors in the near future. A major challenge for the ongoing studies based on massive parallel sequencing remains the discovery and the demonstration of the pathogenic effect of mutations in multicopy spermatogenesis candidate genes.
